# Refractive features and amblyopia in Duane's Retraction Syndrome: A review of the 582 patients^✰^

**DOI:** 10.1016/j.optom.2023.100508

**Published:** 2024-01-12

**Authors:** Masoud Khorrami-Nejad, Mohammad Reza Akbari, Motahhareh Sadeghi, Babak Masoomian, Arash Mirmohammadsadeghi, Mohammed Khalaf Mohsin, Noor Hamad

**Affiliations:** a School of Rehabilitation, Tehran University of Medical Sciences, Tehran, Iran; b Translational Ophthalmology Research Center, Farabi Eye Hospital, Tehran University of Medical Sciences, Tehran, Iran; c Department of Optometry and Optics, Al- Mustaqbal University College, Babylon, Iraq

**Keywords:** Duane retraction syndrome, Refractive error, Amblyopia, Anisometropia

## Abstract

**Purpose:**

To report the visual and refractive characteristics and the prevalence of amblyopia in patients with different types of Duane's Retraction Syndrome (DRS).

**Method:**

This retrospective study was performed on hospital records of 582 DRS patients at Farabi Hospital, Iran, from 2012 to March 2022.

**Results:**

The mean age of patients was 19.4 ± 11.9 (range, 3–70) years [335 (57.6 %) females and 247 (42.4 %) males (*P* < .001)]. DRS type I, II, III, and IV were presented in 347 (59.6 %), 148 (25.4 %), 82 (14.1 %), and 5 (0.9 %) patients, respectively. There were 530 (91.1 %) patients with unilateral and 52 (8.9 %) with bilateral involvement. In the unilateral patients, the DRS eyes' corrected distance visual acuity (CDVA) and astigmatism were significantly worse than the Non-DRS Eyes (*P* < .001). The mean amount of all refractive and visual parameters in bilateral patients' right or left eyes was significantly lower than in unilateral patients' non-DRS eyes (all *P* < .05). Anisometropia was observed in 75(12.9 %) of the patients. Amblyopia was observed in 18.5 % (98 patients) and 36.5 % (19 patients) of unilateral and bilateral DRS patients, respectively (*P* < .001). In unilateral patients, amblyopia was found in 57 (16.4 %) patients with Type I, 22 (14.9 %) patients with Type II, 16 (19.5 %) patients with Type III, and 3 (60 %) patients with Type IV. Forty-four (37.6 %) of patients with amblyopia had anisometropia.

**Conclusion:**

This large-scale study indicates that DRS types differ in terms of refractive error, visual acuity, and the prevalence of amblyopia and anisometropia. Clinicians should be aware of the clinical features associated with different types of DRS.

## Introduction

Duane's Retraction Syndrome (DRS) is a congenital eye movement disorder that mimics inferior oblique or superior oblique muscle overaction. The condition is characterized by restriction in the affected eye's abduction or adduction, co-contraction of the medial and lateral rectus muscles, globe retraction, and the bridle effect of the lateral rectus muscle without vertical muscle dysfunction.^1^ During adduction, the affected eye can appear to upshoot or downshoot.^2^ The average prevalence of DRS among patients with strabismus is between 1 and 5 %.^1^^,^^3^

Numerous theories explain the pathophysiology of DRS. The current theory is that the axons of the third cranial nerve, which innervate the medial rectus (MR) muscle, are misdirected to the lateral rectus (LR) muscle. According to magnetic resonance imaging techniques and autopsy studies, no abducens nerve or nucleus exists, and an aberrant branch of the oculomotor nerve innervates the LR muscle.^4^, ^5^, ^6^ In electromyography evaluation, a decrease in the LR innervation in abduction and a paradoxical increase in adduction have been observed.^7^, ^8^, ^9^

DRS was first described by Stilling and Türk in the late 19th century.^10^ Alexander Duane published a series of 54 patients in 1905, and the syndrome was introduced to the literature and named after him.^2^ Today, despite numerous studies on DRS, the original representation of the syndrome introduced by Duane is the clinically accepted description.^2^ Although there was no classification in the original article, three types of the syndrome were described. Huber was the first scientist who divided DRS into three types in 1974.^11^ Patients with type I, type II, and type III show restriction in abduction, adduction, and both abduction and adduction, respectively. All three types demonstrate globe retraction and narrowing of the palpebral fissure in adduction.^11^ A new subtype of DRS is synergistic divergence or type IV, characterized by a marked limitation of adduction and simultaneous abduction of both eyes on attempted contralateral gaze.^12^

The evaluation of a patient with DRS follows a similar process as for any strabismic patient. This involves examining their vision, performing an objective and subjective refraction, evaluating their eye movements, and sensory and motor fusion, and administering additional tests if necessary. DRS is typically clinically diagnosed through the evaluation of eye movements, measurements in cardinal gaze positions, and forced-duction tests.^13^ Regarding the horizontal eye movement defects, face turn as the compensatory mechanism helps these patients to prevent diplopia and obtain a binocular single vision.^14^ In some patients, small angles of deviation are observed, but mostly binocularity is preserved with a slightly abnormal head posture. However, the presence of a compensatory head posture does not ensure the achievement of binocular fusion, nor does it assure the prevention of amblyopia.^15^ From the clinical perspective, surgical treatment is performed in patients with significant deviation in the primary position or marked abnormal head posture.^16^^,^^17^ However, surgical treatment is not required in patients with no obvious abnormal head posture with almost normal binocular vision and without marked globe retraction.^16^

A considerable body of literature provided evidence for management strategies,^13^^,^^16^, ^17^, ^18^, ^19^, ^20^, ^21^, ^22^, ^23^ manifestations of abnormal head posture^14^^,^^15^^,^^24^ and the other most common clinical finding,^20^^,^^22^^,^^25^, ^26^, ^27^, ^28^, ^29^, ^30^ of patients with DRS. On the other hand, only a few studies have been conducted on the visual and refractive features of DRS patients.^1^^,^^26^^,^^31^, ^32^, ^33^ However, these few studies suffer from relatively small sample sizes or lack of categorization of DRS patients compared to the current study. The present study reviewed the clinical features of 582 patients with different types of DRS. Also, the clinical features in terms of visual acuity, refractive error, and the prevalence of anisometropia and amblyopia were compared between different types of DRS.

## Subjects and methods

This retrospective study involved a review of hospital records from January 2012 to March 2022, focusing on 582 patients diagnosed with DRS at Farabi Eye Hospital in Tehran, Iran. The study received approval from the Institutional Review Board of Tehran University of Medical Sciences (IR.TUMS.FNM.REC.1400.018) and was conducted in accordance with the principles outlined in the Declaration of Helsinki. Informed consent was waived due to the retrospective nature of the study, as per the policy of our University's Ethical Committee. All patients with various types of DRS were included in the study, while exclusion criteria comprised a history of ocular surgeries, a diagnosis of cerebral palsy or developmental delay, and incomplete examination documentation based on medical records.

The collected data from the medical records included information regarding the patient's sex, age, laterality (unilateral or bilateral DRS), best-corrected distance visual acuity (CDVA), type and degree of refractive error, presence of anisometropia, and presence of amblyopia.

The routine ophthalmic examination protocol in our academic center included the following tests: CDVA assessment using the Snellen E-chart; measurement of refraction using the auto-refractometer (Topcon KR-800, Japan); and confirmation of refraction through retinoscopy (Heine Beta 200, Germany). Diagnosis of different types of DRS was made based on assessing adduction or/and abduction limitation, globe retraction, and the presence of overelevation or over-depression in adduction. Type I is defined by marked abduction limitation, normal or mild adduction limitation simultaneous with narrowing palpebral fissure, and globe retraction during adduction and esotropia in the involved eye. Type II is characterized by adduction limitation and exotropia in the affected eye, with normal or slightly limited abduction, narrowing of the palpebral fissure, and globe retraction on adduction. Type III is diagnosed by both adduction and abduction limitation, narrowing of the palpebral fissure, and globe retraction during adduction.^23^^,^^24^ A marked limitation of adduction and simultaneous abduction of both eyes on attempted contralateral gaze defines the synergistic divergence type or type IV.^12^

Myopia was defined as a spherical equivalent of −0.50 D or worse, while hyperopia was defined as a spherical equivalent of +0.50 D or greater.^34^^,^^35^ In patients with unilateral DRS, the prevalence of refractive errors was calculated based on the refractive error of the affected eye, excluding patients with antimetropia. Anisometropia was considered present when there was a difference of −3.00 D or more for myopic eyes, +1.00 D or more for hyperopic eyes, and 1.50 D or more for astigmatic eyes.

The unilateral amblyopic group included patients with CDVA of 20/30 or worse with a difference of more than 2 lines between eyes. Amblyopia was subcategorized as mild (CDVA > 20/50), moderate (CDVA 20/50 to 20/100), and severe (CDVA < 20/100) in the worse eye.^1^ An alternate prism cover test was used to measure the angle of deviation at distance and near fixation. Ocular ductions and versions were assessed in all diagnostics of gazes.

Data analysis was conducted using SPSS version 26 software (IBM, Armonk, NY, USA). Descriptive statistics such as mean, standard deviation, and qualitative representations of ratios and statistical tables were utilized to summarize quantitative data. Subsequently, appropriate statistical tests were employed, including paired *t*-tests to compare quantitative parameters between DRS and non-DRS eyes, and one-way ANOVA to compare quantitative parameters among different types of DRS in both DRS and non-DRS eyes. A significance level of *p* < .05 was used to determine statistical significance.

## Results

The mean age of 582 patients with DRS was 19.4 ± 11.9 (range, 3.0–73.0) years. The study population consisted of 335 (57.6 %) females and 247 (42.4 %) males (*P* < .001). DRS type I, II, III, and IV were presented in 347 (59.6 %), 148 (25.4 %), 82 (14.1 %), and 5 (0.9 %) patients, respectively.

First, we divided patients into unilateral and bilateral groups to better explain the results of this study. Then, visual and refractive features were compared between unilateral and bilateral groups.

### Unilateral Duane retraction syndrome

**Fig. 1:**
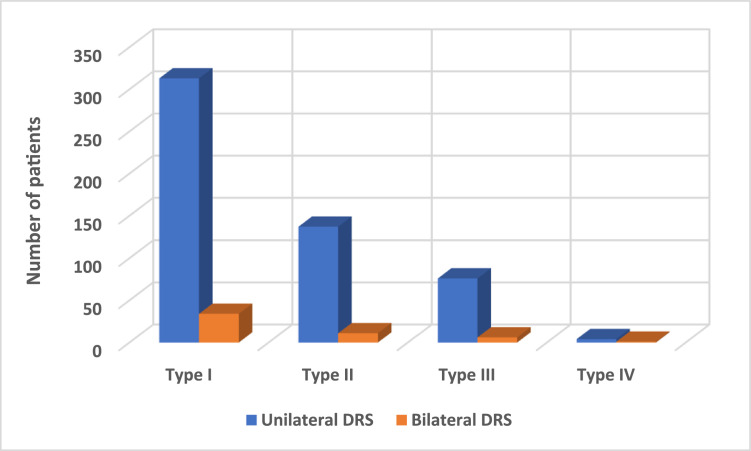
The number of patients classified by laterality and types of Duane retraction syndrome. DRS; Duane retraction syndrome.

The mean age of 530 patients with DRS was 19.4 ± 11.9 (range, 3.0 −73.0) years. There were 312 (58.9 %) females and 218 (41.1 %) males. DRS was found in 391 (73.8 %) left eyes and 139 (26.2 %) right eyes (*P* < .001). Unilateral DRS type I, II, III, and IV were observed in 313 (59.1 %), 137 (25.8 %), 76 (14.3 %), and 4 (0.8 %) patients, respectively. There were 530 (91.1 %) patients with unilateral and 52 (8.9 %) with bilateral involvement. The distribution of patients classified by laterality and types of DRS is shown in Fig. 1.

In all unilateral DRS patients, the CDVA in the DRS eyes was significantly worse than in non-DRS eyes (0.07 ± 0.17 vs. 0.03 ± 0.11 logMAR, *P* < .001). Also, in patients with DRS type I and II, the CDVA in the DRS eyes was significantly worse than in non-DRS eyes (*P* < .001 and *P* = .002, respectively). The comparison of CDVA between DRS and non-DRS eyes in all unilateral and different DRS types is reported in Tables 1 and 2, respectively.

**Table 1:** The comparison of age, CDVA, and refractive error components between DRS and non-DRS eyes in patients with unilateral and bilateral DRS.

Unilateral DRS (*n* = 530)				
		Mean ±SD	Range	*P*-value*
**Age**		19.4 ± 11.9	3.00 to 73.0	
**CDVA (logMAR)**	DRS	0.07 ± 0.17	−1.04 to 1.00	<0.001
	Non-DRS	0.03 ± 0.11	−1.04 to 1.00	
**Sphere (diopter)**	DRS	0.39 ± 2.19	−25.00 to 8.00	.478
	Non-DRS	0.30 ± 1.90	−25.00 to 11.00	
**Cylinder (diopter)**	DRS	−0.70 ± 0.91	−5.00 to 0.75	<0.001
	Non-DRS	−0.52 ± 0.84	−6.50 to 2.50	
**SE (diopter)**	DRS	0.04 ± 2.19	−25.00 to 7.00	.977
	Non-DRS	0.04 ± 1.92	−25.00 to 11.00	

Bilateral DRS (*n* = 52)				

		Mean ±SD	Range	*P*-value

**Age**		19.2 ± 12.3	4.0 to 53.0	–
**CDVA (logMAR)**	Right	0.10 ± 0.17	0.00 to 1.00	.788
	left	0.11 ± 0.16	0.00 to 0.52	
**Sphere (diopter)**	Right	0.94 ± 1.34	−1.25 to 5.50	.770
	left	1.02 ± 1.39	−2.00 to 5.00	
**Cylinder (diopter)**	Right	−0.70 ± 0.71	−3.00 to 0.00	.796
	left	−0.75 ± 1.00	−5.00 to 0.00	
**SE (diopter)**	Right	0.58 ± 1.22	−1.50 to 4.50	.823
	left	0.64 ± 1.33	−3.25 to 5.00	

⁎ Pair *t*-test. DRS; Duane retraction syndrome, CDVA; best-corrected distance visual acuity, n; Number, SE; Spherical equivalent.

**Table 2:** The comparison of age, CDVA, and refractive error components between DRS and non-DRS eyes of unilateral DRS patients.

		Type I (*n* = 313) (Mean ± SD)	Type II (*n* = 137) (Mean ± SD)	Type III (*n* = 76) (Mean ± SD)	Type IV (*n* = 4) (Mean ± SD)	*P*-value*
**Age**		18.3 ± 12.0	20.5 ± 11.9	21.5 ± 11.2	20.0 ± 10.7	.106
**CDVA, (logMAR)**	DRS eye	0.08 ± 0.16	0.07 ± 0.15	0.05 ± 0.19	0.21 ± 0.33	.186
	Non-DRS eye	0.03 ± 0.09	0.02 ± 0.12	0.02 ± 0.15	0.23 ± 0.27	.003
	*P*-value⁎⁎	<0.001	.002	.222	.434	–
**Sphere (diopter)**	DRS eye	0.57 ± 1.59	0.30 ± 2.58	−0.21 ± 3.27	0.19 ± 0.24	.045
	Non-DRS eye	0.49 ± 1.50	0.14 ± 2.44	−0.18 ± 2.18	−0.44 ± 0.88	.023
	*P*-value⁎⁎	.923	.084	.792	.297	–
**Cylinder (diopter)**	DRS eye	−0.67 ± 0.84	−0.72 ± 1.00	−0.77 ± 1.04	−0.88 ± 1.44	.818
	Non-DRS eye	−0.40 ± 0.68	−0.71 ± 0.93	−0.63 ± 1.05	−1.13 ± 1.93	<0.001
	*P*-value⁎⁎	<0.001	.493	.276	.745	–
**Spherical equivalent (diopter)**	DRS eye	0.24 ± 1.62	−0.05 ± 2.56	−0.59 ± 3.23	−0.25 ± 0.84	.030
	Non-DRS eye	0.30 ± 1.50	−0.22 ± 2.44	−0.49 ± 2.22	−1.33 ± 2.10	<0.001
	*P*-value⁎⁎	.413	.118	.658	.241	–

⁎ One-way ANOVA.

⁎⁎ Pair *t*-test. DRS; Duane retraction syndrome, CDVA; best-corrected distance visual acuity, N; Number.

**Fig. 2:**
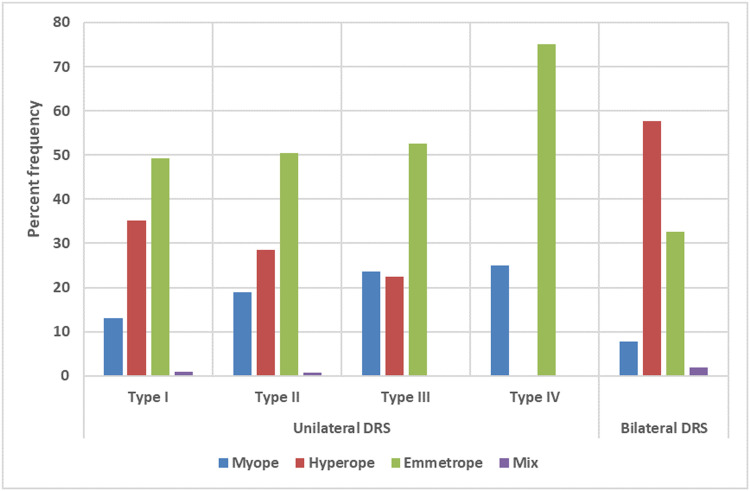
The percent frequency of different types of refractive error among patients with different types of Duane retraction syndrome.

In all unilateral DRS patients, hyperopia was the most common type of refractive error found in 166 (31.3 %), followed by myopia (*n* = 86, 16.2 %) and antimetropia (*n* = 4, 0.8 %). The most prevalent type of refractive error in DRS types I and II was hyperopia, but in DRS types III and IV was myopia. The prevalence of hyperopia was significantly higher in DRS type I (*n* = 110, 35.1 %) followed by type II (*n* = 39, 28.5 %), and type III (*n* = 17, 22.4 %) (all *P* < .05). On the other hand, the prevalence of myopia was significantly higher among DRS type III (*n* = 18, 23.7 %) followed by type II (*n* = 26, 19.0 %), and type I (*n* = 41, 13.1 %) (all *P* < .05). The percent frequency of different types of refractive error among patients with different types of DRS is reported in Fig. 2.

The mean cylindrical power in the DRS eye was significantly higher than in non-DRS eyes (−0.70 ± 0.91 vs. −0.52 ± 0.84 diopter, *P* < .001). Regarding the comparison of astigmatism in different DRS types, astigmatism only in the DRS type I eye was significantly higher than in non-DRS eyes (*P* < .001), and in other DRS types, there were no significant differences between the eyes (*P* > .05). Although the mean sphere and spherical equivalent did not exhibit significant differences between DRS and non-DRS eyes (*P* > .05), these components in the DRS eyes of patients with type I were significantly higher than in the DRS eyes of patients with types II and III and *P* = .030), respectively. The comparison of refractive error components between DRS and non-DRS eyes classified by laterality and DRS types are reported in Tables 1 and 2, respectively.

In all unilateral DRS, anisometropia was found in 63 (11.9 %) patients, consisting of 34 (10.9 %) in type I, 21 (15.3 %) in type II, and 8 (10.5 %) in type III. There was no patient with anisometropia in DRS type IV. The prevalence of anisometropia in the DRS type II (15.3 %) was significantly higher than in other groups (*P* < .05). Regarding the types of anisometropia, there were 47 (8.9 %) anisohyperopic patients, 13 (2.5 %) patients with anisoastigmatism, 2 (0.4 %) anisomyopic patients, and 1(0.2 %) patient with antimetropia. The prevalence of anisohyperopia was significantly higher than in other anisometropic types (*P* < .001).

**Fig. 3:**
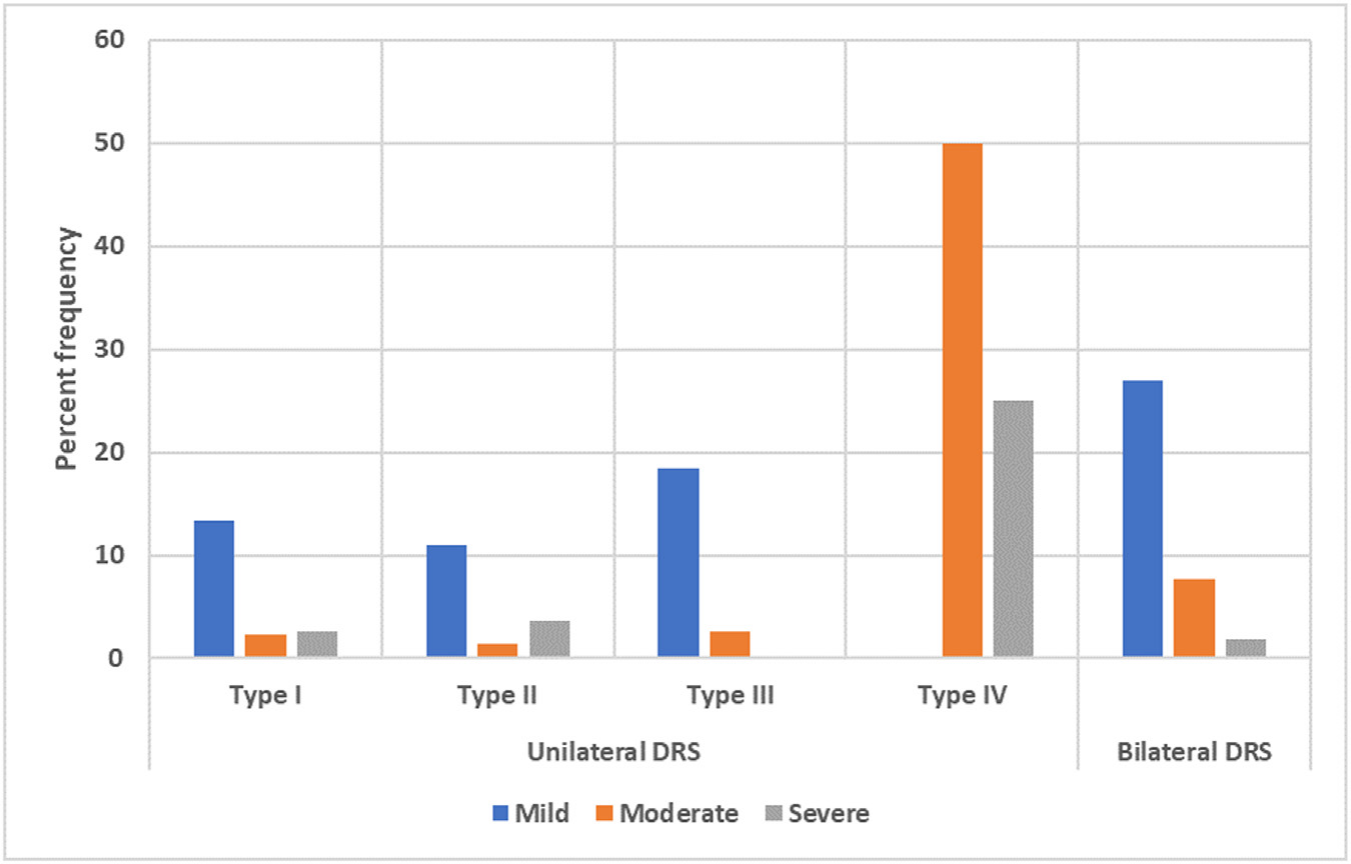
The percent frequency of different severity of amblyopia in the unilateral and bilateral DRS. DRS; Duane retraction syndrome.

Amblyopia was observed in 98 (18.5 %) patients who had unilateral DRS, which consisted of 57 (16.4 %) patients with Type I, 22 (14.9 %) patients with Type II, 16 (19.5 %) patients with Type III, and 3 (60 %) patients with Type IV. In patients with unilateral DRS type I, II, and III, the most frequent severity of amblyopia was mild. The percent frequency of different severities of amblyopia in unilateral DRS patients is shown in Fig. 3. Strabismic amblyopia was the most common type of amblyopia found in 62 (11.7 %) patients, followed by combined-mechanism amblyopia in 31 (5.8 %) patients and refractive amblyopia in 5 (0.9 %) patients. Thirty-six (36.7 %) of the patients with amblyopia had anisometropia, including 31 (31.6 %) anisohyperopic, 4 (4.1 %) anisoastigmatism and 1 (1.0 %) patient with antimetropia.

### Bilateral Duane retraction syndrome

The mean age of 52 patients with bilateral DRS was 19.2 ± 12.3 (range, 4.0 to 53.0) years. There were 23 (44.2 %) females and 29 (55.8 %) males. The most frequent type of bilateral DRS was type I, observed in 34 (65.4 %) patients, followed by type II (*n* = 11, 21.2 %), type III (*n* = 6, 11.5 %), and type IV (*n* = 1, 1.9 %) patient, respectively. The distribution of patients classified by laterality and types of DRS is shown in Fig. 1.

In all bilateral DRS patients, the CDVA in the right and left DRS eyes was 0.10 ± 0.17 and 0.11 ± 0.16 logMAR, respectively (*P* = .788).

Hyperopia was the most common type of refractive error, found in 30 (57.7 %) patients. The percent frequency of different types of refractive error among patients with different types of DRS is illustrated in Fig. 2. There were no significant differences in different refractive error components between the right and left eyes (*P* > .05).

In bilateral DRS patients, anisometropia was found in 12 (23.1 %) patients, consisting of 11 (21.2 %) with anisohyperopia and 1 (1.9 %) patient with anisoastigmatism. The prevalence of amblyopia in bilateral DRS patients was 36.5 % (19 patients). The most frequent severity of amblyopia was mild, found in 14 (26.9 %) patients. The percent frequency of different severities of amblyopia in the bilateral DRS is shown in Fig. 3.

The most prevalent type of amblyopia was strabismic amblyopia, observed in 11 (21.2 %) patients, followed by combined-mechanism amblyopia in 7 (13.5 %) patients and refractive amblyopia in 1 (1.9 %) patient. Eight patients with amblyopia had anisometropia, including seven with anisohyperopia and one with anisoastigmatism.

Regarding comparison of unilateral and bilateral DRS patients, the mean amount of sphere and spherical equivalent in either eye of bilateral patients was significantly higher than in both DRS and non-DRS eyes of unilateral patients (all *P* < .05). Also, the CDVA in either eye of bilateral patients was significantly lower than in both eyes of unilateral patients (all *P* < .05). In addition, the prevalence of amblyopia in bilateral DRS (36.5 %) was significantly higher than unilateral patients (18.5 %) (*P* < .001).

In all DRS patients, anisometropia was observed in 75 (12.9 %) patients, including 58 (10.0 %) anisohyperopic patients, 14 (2.4 %) patients with anisoastigmatism, 2 (0.3 %) anisomyopic patients, and 1(0.2 %) patient with antimetropia. Also, anisometropia was found in 41 (11.8 %) patients with type I, 24 (16.2 %) patients with type II, and 10 (12.2 %) patients with type III. In terms of amblyopia in all patients with DRS, 117 (20.1 %) patients had amblyopia consisting of 85 (14.6 %) mild, 17 (2.9 %) moderate, and 15 (2.6 %) severe amblyopia. Strabismic amblyopia was the most frequent type of amblyopia found in 73 (62.4 %) patients, followed by combined-mechanism amblyopia in 38 (32.5 %) patients and refractive amblyopia in 6 (5.1 %) patients. Forty-four (37.6 %) of the patients with amblyopia had anisometropia, including 38 (32.5 %) anisohyperopic, 5 (4.3 %) anisoastigmatism and 1 (0.9 %) patient with antimetropia.

## Discussion

DRS is the most common type of strabismus among congenital retraction syndromes.^20^ DRS is clinically well-defined, with narrowing of the palpebral fissure on adduction and globe retraction, as well as limitation of abduction and adduction of the affected eye, often with varying amounts of adduction limitation.^11^ Previous studies have reported DRS is more common in females than males, and more common in the left eye than in the right.^1^^,^^25^^,^^26^ Our findings concurred as we also found a higher proportion of DRS patients were female 335 (57.6 %) than male 247 (42.4 %) and the prevalence of DRS in the left eye (73.8 %) was significantly higher than in the right eye (26.2 %). This condition can be explained by sex-related genetic transmission patterns and an excess of dominant usage of one half of the brain.^19^^,^^28^

DRS is accompanied by different types of refractive errors at varying degrees. Zhang et al. reported that emmetropia was found in 50 %, hyperopia in 35.6 %, myopia in 11 %, and astigmatism in 0.84 % of patients.^36^ Isenberg and Urist reported 67 % hyperopia, 18 % myopia, and 16 % emmetropia.^30^ In terms of refractive error in unilateral DRS patients, however, we found that the prevalence of hyperopia in the DRS type I was significantly higher than in type II, and in type II was significantly higher than in type III. Also, the most frequent type of refractive error in bilateral DRS patients was hyperopia, found in 30 (57.7 %) patients. In line with our findings, Anvari et al. reported that hyperopia was the most prevalent type of refractive error among patients with DRS. The authors noted that 9 (8.7 %), 59 (56.7 %), and 48 (37.2 %) patients had myopia, hyperopia, and cylindrical refractive error, respectively.^27^ The distribution of refractive errors observed in the present study was comparable to that noted by most other studies on DRS patients.^27^^,^^30^^,^^36^^,^^37^ In terms of cylindrical refractive error in patients with unilateral DRS, we realized that the mean cylindrical value in the DRS eye was significantly higher than in non-DRS eyes. This finding might be due to the co-contraction of LR and MR muscles and globe retraction during adduction.

Another key finding of the current study was that the CDVA in the DRS eyes was significantly worse in the unilateral patients than in non-DRS eyes. In our study, the prevalence of anisometropia was 11.8 % in type I, 16.2 % in type II, and 12.2 % in type III. No patients with DRS type IV showed anisometropia. The prevalence of anisometropia in the DRS type II was significantly higher than in other groups.

In our study, anisometropia and amblyopia were observed in 75 (12.9 %) and 117 (20.1 %) patients, respectively. In line with our study, Duane reported in 1905 that most of his patients were hyperopic, with the rate of amblyopia being 21 %.^2^ In Kirkham's series, the rate of amblyopia was reported as 25 %, and anisometropia was found in half of the patients.^32^ In another study, Zhang et al. reported this rate as 33.8 %.^36^ In a case series of 101 patients with DRS, Mehel et al. found that the rate of amblyopia was 32.9 %.^28^ The reported prevalence of amblyopia was approximately the same in other studies.^38^ In addition to the studies mentioned, it is noteworthy that the prevalence of amblyopia observed in our study among patients with DRS falls within the range reported in other relevant studies.^21^^,^^23^^,^^28^^,^^29^^,^^39^^,^^40^

Nevertheless, some studies reported a lower rate of amblyopia. For example, Tredici and von Noorden reported that the prevalence of anisometropia and amblyopia in patients with DRS was 17 % and 3 %, respectively.^31^ They asserted that anisometropia and amblyopia are not more common in DRS than in the general population. Maruo et al. also detected amblyopia in 2 cases in their series of 266 patients (0.08 %).^18^

Furthermore, our findings revealed that the prevalence of amblyopia differed significantly between unilateral and bilateral DRS patients, with rates of 18.5 % and 36.5 %, respectively. These results are consistent with previous studies that have also reported a higher prevalence of amblyopia in bilateral DRS compared to unilateral cases.^1^^,^^30^^,^^32^^,^^41^

Moreover, we found that in unilateral patients, the prevalence of amblyopia in DRS type I, type II, type III, and type IV was 16.4 %, 14.9 % and 19.5 %, respectively. In concurrence with our study, Muhan et al. found no significant difference in the prevalence of amblyopia among the various types of DRS.^26^ But according to Tomac et al., patients with type 2 and type 3 DRS were significantly more likely to have amblyopia.^40^

In our study, we found that the most common severity of amblyopia was mild. Our findings are consistent with those of Anvari et al., who also reported mild amblyopia (43.8 %) as the most prevalent type.^27^ However, in contrast to our study, Kekunnaya et al. reported severe and moderate amblyopia as the most common severities.^19^

In our study, strabismic amblyopia was the most frequent type of amblyopia found in 73 (62.4 %) patients. Like our study, manifest strabismus is a more determinant factor than anisometropia in the development of amblyopia in other studies. DeRespinis et al. reported that amblyopia in DRS patients was mainly due to strabismus and not anisometropia.^23^ Also, Mohan et al. reported that in DRS type I, the most common type of amblyopia was strabismic amblyopia. They further noted that in DRS type II and III, the only source of amblyopia was strabismus, and there were no patients with anisometropic amblyopia.^26^

From the clinical perspective, DRS patients with strabismus habitually turn their head to compensate the deviation and thereby can achieve binocular single vision.^14^^,^^19^ Amblyopia may develop in patients who are unable to correct for the misalignment with head turn. However, Turan et al. reported that 22.2 % of patients with abnormal head posture did not have stereopsis and flat fusion.^15^ In addition, in a recently published paper, abnormal head posture was detected in 75.5 % of DRS patients and the prevalence of amblyopia in DRS patients with abnormal head posture (15.3 %) was significantly lower than in DRS patients with a normal head posture (22.5 %). But it is notable that 15.3 % of DRS patients with abnormal head posture still had amblyopia.^17^ Therefore, the presence of AHP cannot guarantee binocular fusion and prevent strabismic amblyopia because The prevalence of amblyopia remains relatively high in patients with abnormal head posture.

## Conclusions

In summary, in unilateral cases, DRS eyes had significantly worse CDVA compared to non-DRS eyes. Hyperopia was the most common type of refractive error in unilateral DRS, with a higher prevalence in type I than in type II. The mean cylindrical power was higher in DRS eyes compared to non-DRS eyes, and astigmatism was significantly higher in type I. Anisometropia was found in 12.9 % of patients and was most common in DRS type II. Amblyopia was found in 20.1 % of patients and strabismus was the most common cause of amblyopia. The prevalence of amblyopia was significantly higher in bilateral DRS patients than in unilateral cases. Overall, this research provides valuable insights into the visual and refractive characteristics of DRS patients, highlighting the importance of carefully assessing patients for amblyopia. The findings of this large-scale study indicated that different DRS types are associated with different visual acuity, refractive errors, and prevalence/severity of amblyopia. Clinicians should be aware of the clinical features associated with different DRS types.

## Conflicts of interest

The authors have no conflicts of interest to declare.
